# Iron Overload Accelerates the Progression of Diabetic Retinopathy in Association with Increased Retinal Renin Expression

**DOI:** 10.1038/s41598-018-21276-2

**Published:** 2018-02-14

**Authors:** Kapil Chaudhary, Wanwisa Promsote, Sudha Ananth, Rajalakshmi Veeranan-Karmegam, Amany Tawfik, Pachiappan Arjunan, Pamela Martin, Sylvia B. Smith, Muthusamy Thangaraju, Oleg Kisselev, Vadivel Ganapathy, Jaya P. Gnana-Prakasam

**Affiliations:** 1 Department of Medicine, Washington University, St. Louis, Missouri USA; 2 National Institute of Health, Bethesda, Maryland USA; 3 Department of Biochemistry and Molecular Biology, Medical College of Georgia, Augusta University, Augusta, Georgia USA; 4 Dental College of Georgia, Augusta University, Augusta, Georgia USA; 5 Department of Cellular Biology and Anatomy, Medical College of Georgia, Augusta University, Augusta, Georgia USA; 6 Department of Cell Biology and Biochemistry, Texas Tech University Health Sciences Center, Lubbock, Texas USA; 7 Department of Ophthalmology and Department of Biochemistry & Molecular Biology, Saint Louis University, St. Louis, Missouri USA

## Abstract

Diabetic retinopathy (DR) is a leading cause of blindness among working-age adults. Increased iron accumulation is associated with several degenerative diseases. However, there are no reports on the status of retinal iron or its implications in the pathogenesis of DR. In the present study, we found that retinas of type-1 and type-2 mouse models of diabetes have increased iron accumulation compared to non-diabetic retinas. We found similar iron accumulation in postmortem retinal samples from human diabetic patients. Further, we induced diabetes in HFE knockout (KO) mice model of genetic iron overload to understand the role of iron in the pathogenesis of DR. We found increased neuronal cell death, vascular alterations and loss of retinal barrier integrity in diabetic HFE KO mice compared to diabetic wildtype mice. Diabetic HFE KO mouse retinas also exhibited increased expression of inflammation and oxidative stress markers. Severity in the pathogenesis of DR in HFE KO mice was accompanied by increase in retinal renin expression mediated by G-protein-coupled succinate receptor GPR91. In light of previous reports implicating retinal renin-angiotensin system in DR pathogenesis, our results reveal a novel relationship between diabetes, iron and renin-angiotensin system, thereby unraveling new therapeutic targets for the treatment of DR.

## Introduction

Diabetic retinopathy (DR) is a chronic progressive complication associated with prolonged hyperglycemia in diabetes mellitus. DR is the most common microvascular complication in diabetic patients that can progress to the loss of vision^1^. DR is characterized by inflammation, neurodegeneration and microvascular alterations in the retina^2–5^. The most common cause of vision loss depends on the type of diabetes, with type 1 diabetes presenting as proliferative retinopathy causing severe hemorrhage in the vitreous^6^, and with type 2 diabetes manifesting as macular edema caused by breakdown of the blood-retinal barrier^7^. Oxidative stress is considered to play an important role in the pathogenesis of DR^8^.

In the retina, there are many iron-containing proteins that are involved in the phototransduction cascade. Although an essential micronutrient for the function of many proteins, iron is a potentially harmful pro-oxidant when present in large quantities. Excess iron can undergo Fenton reaction, catalyzing the conversion of H_2_O_2_ to hydroxyl radical, which is considered the most reactive oxygen species. Hydroxyl radicals cause lipid peroxidation, DNA strand breaks, and degradation of cellular components leading to tissue damage^9^. Hence stringent mechanisms maintain iron levels by regulating proteins involved in iron homeostasis. Dysregulation of local iron homeostasis has been shown to play a role in the etiology of several neurodegenerative disorders like Parkinson’s, Alzheimer’s and amyotrophic lateral sclerosis^10–13^. Similarly, abnormal iron deposits have been associated with ocular diseases such as age-related macular degeneration, cataracts and glaucoma^14–16^. In addition, there are clinical reports on positive link between iron levels and proliferative retinopathies^17–22^, however, implications of the retinal iron imbalance in the pathogenesis of DR has not been elucidated.

Renin-angiotensin system (RAS) plays an important role in the control of blood pressure and electrolyte homeostasis. The enzyme Pro/renin cleaves its substrate, angiotensinogen, to form angiotensin I. Angiotensin converting enzyme (ACE) converts angiotensin I to angiotensin II, a potent vasoconstrictor and a stimulant of aldosterone release. Angiotensin II synthesis in numerous tissues and organs has demonstrated the presence of tissue-based RAS that are independent of circulating RAS^23^. Retina contains all the elements of RAS and intraocular Angiotensin II formation has been shown to be independent of the circulating RAS^24^. The tissue RAS acts in a paracrine/autocrine manner to regulate organ function and is involved in the pathologic events leading to end-organ damage. The retinal RAS has also been implicated in DR pathogenesis^25^. The Diabetic Retinopathy Candesartan Trials (DIRECT) reported that angiotensin receptor blocker (ARB) candesartan reduced retinopathy development in normotensive normoalbuminuric diabetic patients without DR but not in patients with mild to moderate DR^26,27^. The Renin-Angiotensin System Study (RASS) found significant delay in the progression of retinopathy in diabetic patients treated with the angiotensin-converting-enzyme inhibitor (ACEI)^28^.

GPR91 is a G-protein–coupled receptor for succinate^29^. Imbalance in the local tissue energy demand and supply leads to the citric acid cycle intermediate succinate, which is normally present within the mitochondria, to be released into the extracellular medium. The extracellular succinate serves as an agonist for GPR91^30^. In the kidney, succinate-induced activation of GPR91 has been shown to regulate the expression of renin-angiotensin system^31,32^. GPR91 is expressed in retinal ganglion cells and retinal pigment epithelium^33,34^. We have reported previously that retinal iron overload induces GPR91 expression in the retinas of HFE and hemojuvelin knockout mice models of hemochromatosis, a genetic disorder of iron overload, and subsequently stimulates the production of downstream vascular endothelial growth factor (VEGF)^34,35^. HFE is an important iron regulatory protein that senses cellular iron status and regulates iron uptake by competitively inhibiting transferrin receptor. We have previously reported that HFE is expressed predominantly in the basolateral membrane of the retinal pigment epithelium, and HFE knockout (KO) mice accumulate iron in the retina with significant retinal degeneration by 18 months of age^36^. In the present study, we induced diabetes in HFE knockout (KO) mice to understand the mechanistic role of iron, GPR91 and renin angiotensin system in the pathogenesis of DR.

## Results

### Increased iron accumulation in diabetic retina

**Figure 1:**
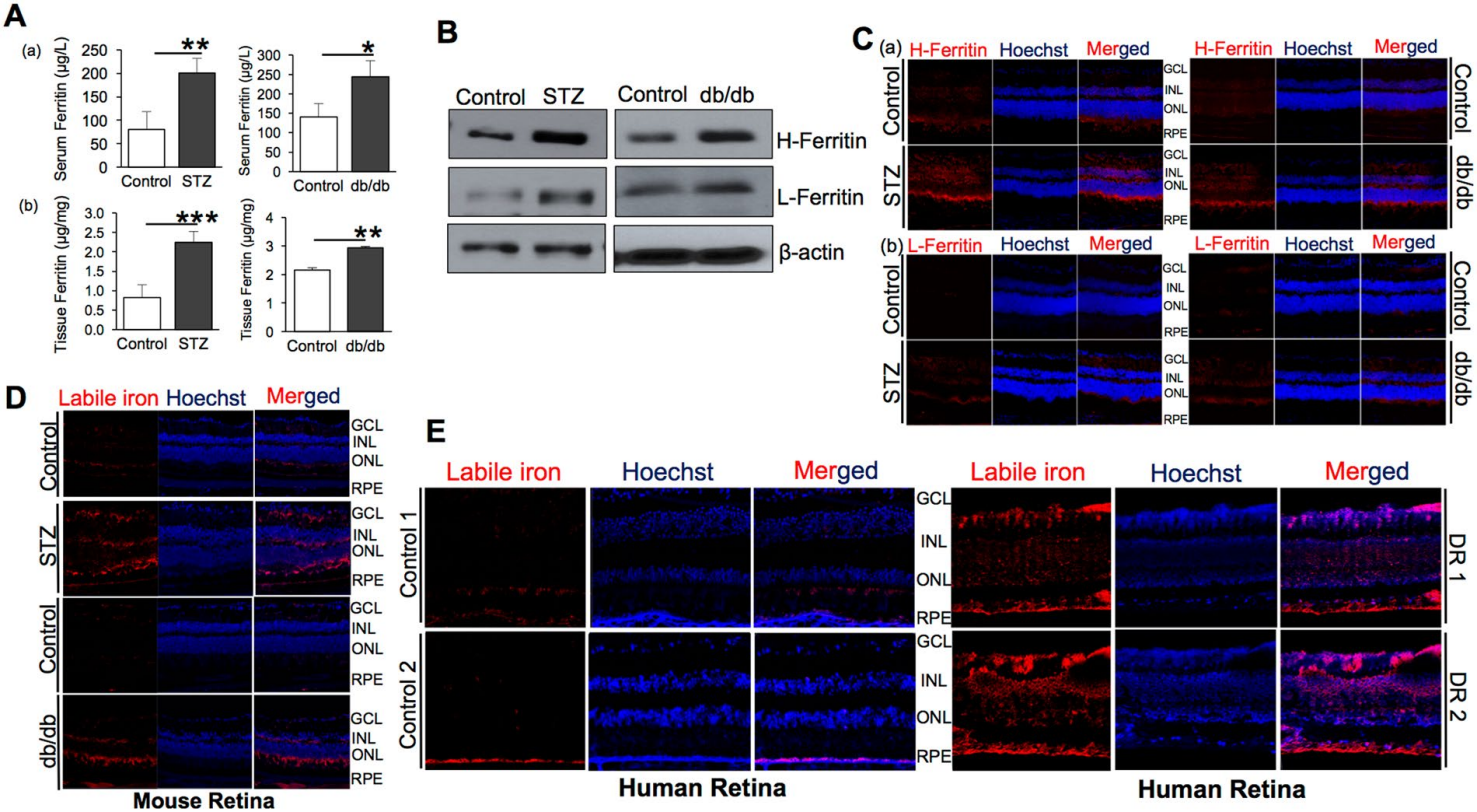
Iron accumulation in diabetic retina. (**A**) Quantification of ferritin levels in the (a) serum and (b) eye tissues of 12-week type 1 (STZ) and type 2 (db/db) diabetic mice by ELISA. (**B**) Heavy chain H-ferritin and light chain L-ferritin levels in the retinas of type 1 (STZ) and type 2 (db/db) diabetic mice models by western blot. Blots cropped from different parts of the same gel or from different gels are separated by white space. (**C**) Representative images of retinal sections from control vs type 1 (STZ) and type 2 (db/db) diabetic mice immunostained for (**a**) heavy chain H-ferritin and (**b**) light chain L-ferritin. (**D**) Labile iron (Fe^2+^) in retinal tissues of control vs type 1 (STZ) and type 2 (db/db) 12-week diabetic mice were detected using FeRhoNox-1 fluorescent imaging probe. (**E**) Representative images of retinal sections from normal and diabetic human patients (Diabetic retinopathy, DR) stained for labile iron (Fe^2+^) using FeRhoNox-1 fluorescent imaging probe. GCL-Ganglion Cell Layer, INL-Inner Nuclear Layer, ONL-Outer Nuclear Layer, RPE-Retinal Pigment Epithelium. *p < 0.05; **p < 0.001; ***p < 0.0001.

Iron levels in serum and eye tissues were measured in type 1 and type 2 diabetic mouse models by quantifying ferritin, an iron-storage protein, which is upregulated during intracellular iron accumulation. Type 1 diabetes was induced in C57BL/6 mice by intraperitoneal injection of streptozotocin (STZ). BKS.Cg Lepr^db^/J (db/db) mice model was used for type 2 diabetes. Ferritin levels were found to be significantly upregulated in the serum and eye tissues of type 1 and 2 diabetic mice compared to non-diabetic control mice using ELISA (Fig. 1A). Western blot (Fig. 1B) and immunofluorescence (Fig. 1C) showed upregulation of heavy chain (H-) and light chain (L-) ferritin levels in the retina of type 1 (STZ) and type 2 (db/db) diabetic mice. Labile iron staining using FeRhoNox-1 fluorescent imaging probe confirmed increased Fe^2+^ iron accumulation in the diabetic mice (Fig. 1D) and the diabetic human (Fig. 1E) retinal sections demonstrating that retina accumulates iron during diabetes.

### Increased outer and inner retinal barrier dysfunction in diabetic mice with iron overload

**Figure 2:**
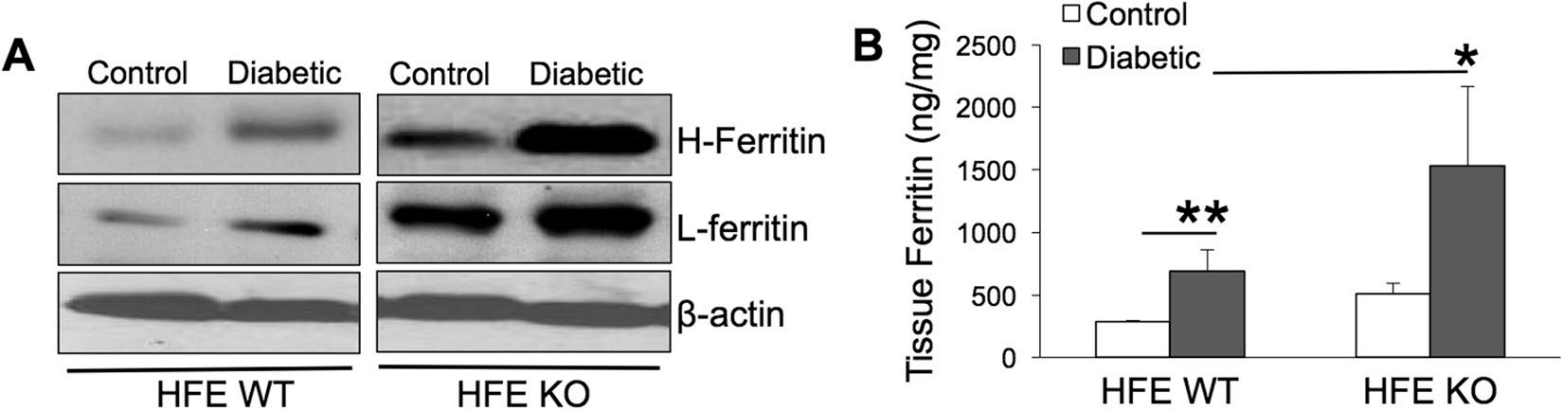
Ferritin expression is upregulated in the retina of diabetic HFE KO mice model of iron overload. (**A**) Western blot for Heavy chain (H-) and Light chain (L-) Ferritin in the retina of non-diabetic control and 16-week diabetic HFE WT and KO mice. β-actin was used as a loading control. Blots cropped from different parts of the same gel or from different gels are separated by white space. (**B**) ELISA for ferritin in the retina of non-diabetic and diabetic HFE WT and KO mice. *p < 0.05; **p < 0.001.

To understand if iron accumulation alters the pathogenesis of diabetic retinopathy, we induced diabetes in HFE KO mice, a genetic model of iron overload, using STZ. We reported previously that HFE KO mice have increased retinal iron accumulation with age-dependent retinal degeneration^36,37^. In the present study, we confirmed that retinal iron accumulation was 2-fold higher in HFE KO diabetic mice compared to HFE WT diabetic mice as indicated by ferritin upregulation in the eye tissues of diabetic HFE KO mice using western blot and ELISA (Fig. 2A,B).

**Figure 3:**
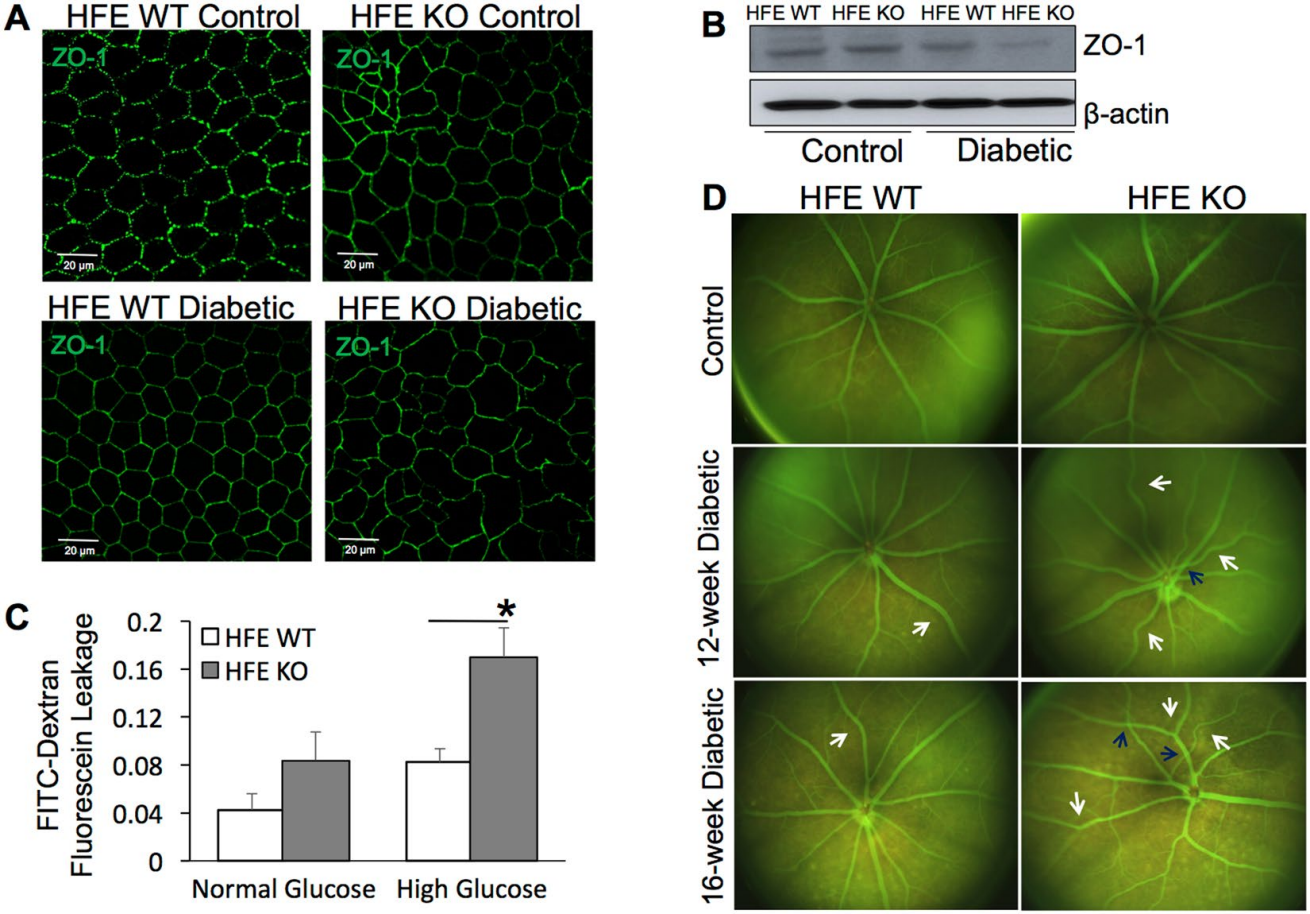
Altered outer and inner retinal barrier function in diabetic HFE KO mice. (**A**) Tight junction protein Zonula occludin-1 (ZO-1) immunofluorescence in retinal pigment epithelium (RPE) flat mounts prepared from non-diabetic control and 16-week post-diabetic HFE WT and KO mice. (**B**) Western blot analysis of ZO-1 expression in protein extracted from the RPE of non-diabetic and diabetic HFE WT and KO mice. β-actin was used as a loading control. Blots cropped from different parts of the same gel or from different gels are separated by white space. (**C**) FITC-Dextran (40-kD) trans epithelial permeability assay performed on confluent monolayers of primary RPE cells established from HFE WT and HFE KO mouse eyes grown in normal glucose (5.5 mM) or high glucose (25 mM). Data are presented as mean ± S.E. *p < 0.01. (**D**) Fluorescein angiography and fundoscopic imaging of non-diabetic control vs diabetic HFE WT and KO mice.

Maintaining the two components of blood retinal barrier (BRB), outer RPE cell and inner endothelial cell integrity, is quintessential for retinal health. Alterations of the BRB play a crucial role in the development of DR^38^. To evaluate if iron overload during diabetes affects barrier integrity, we prepared RPE flatmounts from control and diabetic HFE WT and KO mouse eyes and stained for the tight-junction protein zona occludens-1 (ZO-1) to assess outer retinal barrier integrity (Fig. 3A). There was no significant difference in ZO-1 staining between non-diabetic HFE WT and KO control mice (Fig. 3A, top panel). ZO-1 labeled flat-mount preparations from 12-week post diabetic HFE WT mice retained the characteristic RPE phenotype showing a monolayer of uniformly stained cobblestone-shaped RPE cells (Fig. 3A, bottom left panel). On the contrary, RPE flat mounts from 12-week post diabetic HFE KO mice had hypertrophied RPE cells of abnormal shape along with a disruption in the continuity of ZO-1 staining in some areas (Fig. 3A, bottom right panel). For quantitative confirmation of ZO-1 labeling observed in immunostained flat-mounts, western blot analysis was performed with proteins extracted from the RPE of diabetic and non-diabetic HFE WT and KO mice. ZO-1 protein expression was significantly reduced in diabetic HFE KO mice compared to diabetic HFE WT mice with no significant difference in ZO-1 protein levels between non-diabetic HFE WT and KO control (Fig. 3B). To determine if disruption of the tight junction in turn affects barrier integrity, primary RPE cells were prepared from HFE WT and KO mouse eyes and cultured for 6 weeks in the presence of normal glucose (5.5 mM) or high glucose (25 mM) on permeable transwell for the formation of confluent monolayer. The apical-to-basolateral movement of 40- kD FITC-Dextran dye was measured as an indicator of transepithelial permeability (Fig. 3C). The increase in the accumulation of dye in the basolateral compartment of HFE KO RPE cells grown in high glucose condition indicates that these cells were significantly more permeable to FITC- Dextran than HFE WT RPE cells grown in high glucose condition. These findings for the first time implicates retinal iron overload directly to disruption of BRB.

Retinas of non-diabetic control and diabetic HFE WT and KO mice were evaluated using Micron III *in-vivo* retinal imaging microscope. Fundoscopic imaging of non-diabetic HFE WT and KO mice demonstrated no signs of abnormality, and the retinas of these animals appeared to be healthy. Simultaneous fluorescein angiography of these retinas showed normal retinal vasculature (Fig. 3D, top panel). In contrast, fundoscopic imaging and fluorescein angiography of diabetic HFE WT and KO mice showed increase in pigmentary and vascular abnormalities. At 8-weeks post diabetes, WT diabetic eyes showed no signs of retinal pathology. However, vascular anomalies were detected frequently early on in HFE KO diabetic eyes. By 12- weeks (Fig. 3D, middle panel) and 16-weeks post diabetes (Fig. 3D, bottom panel), the incidence of tortuous vessels, arteriovenous crossings and retinal vein occlusion with associated pigmentary abnormalities were much higher in diabetic HFE KO mice in comparison to diabetic HFE WT mice.

### Increased neuronal cell loss in retina of diabetic mice with iron overload

**Figure 4:**
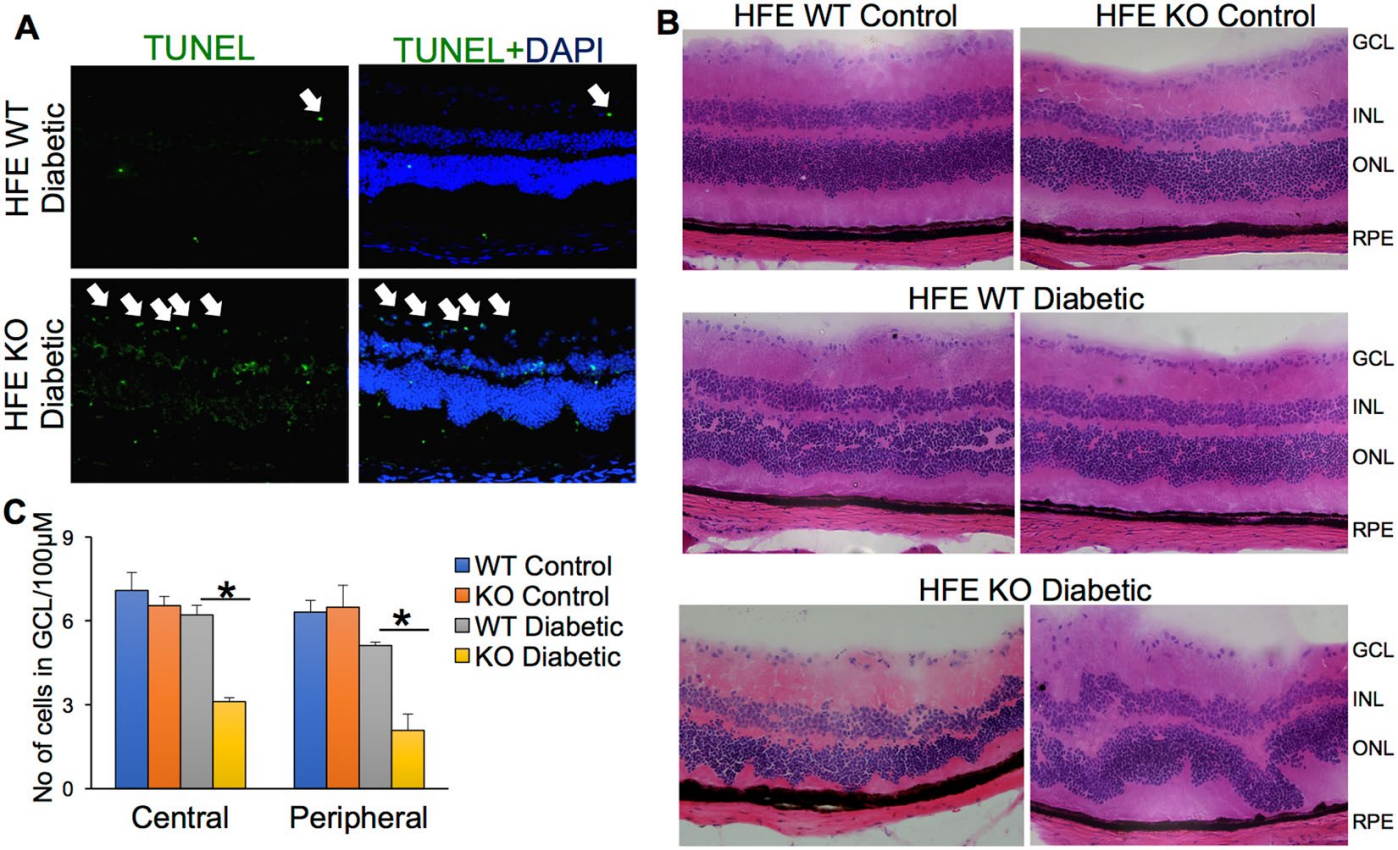
Accelerated neuronal cell loss and morphological changes in retina of diabetic HFE KO mice. (**A**) TUNEL assay in the eye sections of diabetic HFE WT and KO mice. Green signals indicate apoptotic cells. (**B**) Hematoxylin and eosin stained retinal sections from non-diabetic HFE WT and KO mice (top panel) compared to diabetic HFE WT (middle panel) and diabetic HFE KO mice (bottom panel) (**C**) Quantification of number of cells in ganglion cell layer in the central and peripheral regions of retinal sections from non-diabetic and diabetic HFE WT and KO mice. *p < 0.01.

To determine if retinal iron accumulation during diabetes causes apoptosis of retinal cells, we performed TUNEL assay in eye sections from the 12-week diabetic HFE WT and KO mice. The cells with significantly higher apoptosis were found in the ganglion cell layer (GCL) of diabetic HFE KO mice in comparison to diabetic HFE WT mice. After GCL, there was considerable increase in apoptotic cells in the inner nuclear layer (INL), outer nuclear layer and to a lesser extent in the RPE of diabetic HFE KO mice compared to diabetic HFE WT mice (Fig. 4A). We did not find any apoptotic cells in the non-diabetic HFE WT and KO control (data not shown). Hematoxylin and eosin stained retinal sections from non-diabetic WT and KO mice showed normal morphology (Fig. 4B, top panel). While 16-week post diabetic HFE WT mice had mild cell loss in the GCL and INL (Fig. 4B, medium panel), sections from diabetic HFE KO mice showed significant loss of cells in GCL and INL, with focal points where photoreceptor cells migrated towards RPE layer (Fig. 4B, bottom panel). Since we found maximum apoptosis and cell loss in GCL, we quantified the average number of cells present in GCL per 100 μm in central and peripheral retina. GCL of diabetic HFE KO mice had 50% reduction in cell number compared to diabetic HFE WT mice both in the central and peripheral retina with no difference in cell number between non-diabetic HFE WT and KO control retina (Fig. 4C).

### Increased oxidative stress and inflammation in diabetic mice with iron overload

**Figure 5:**
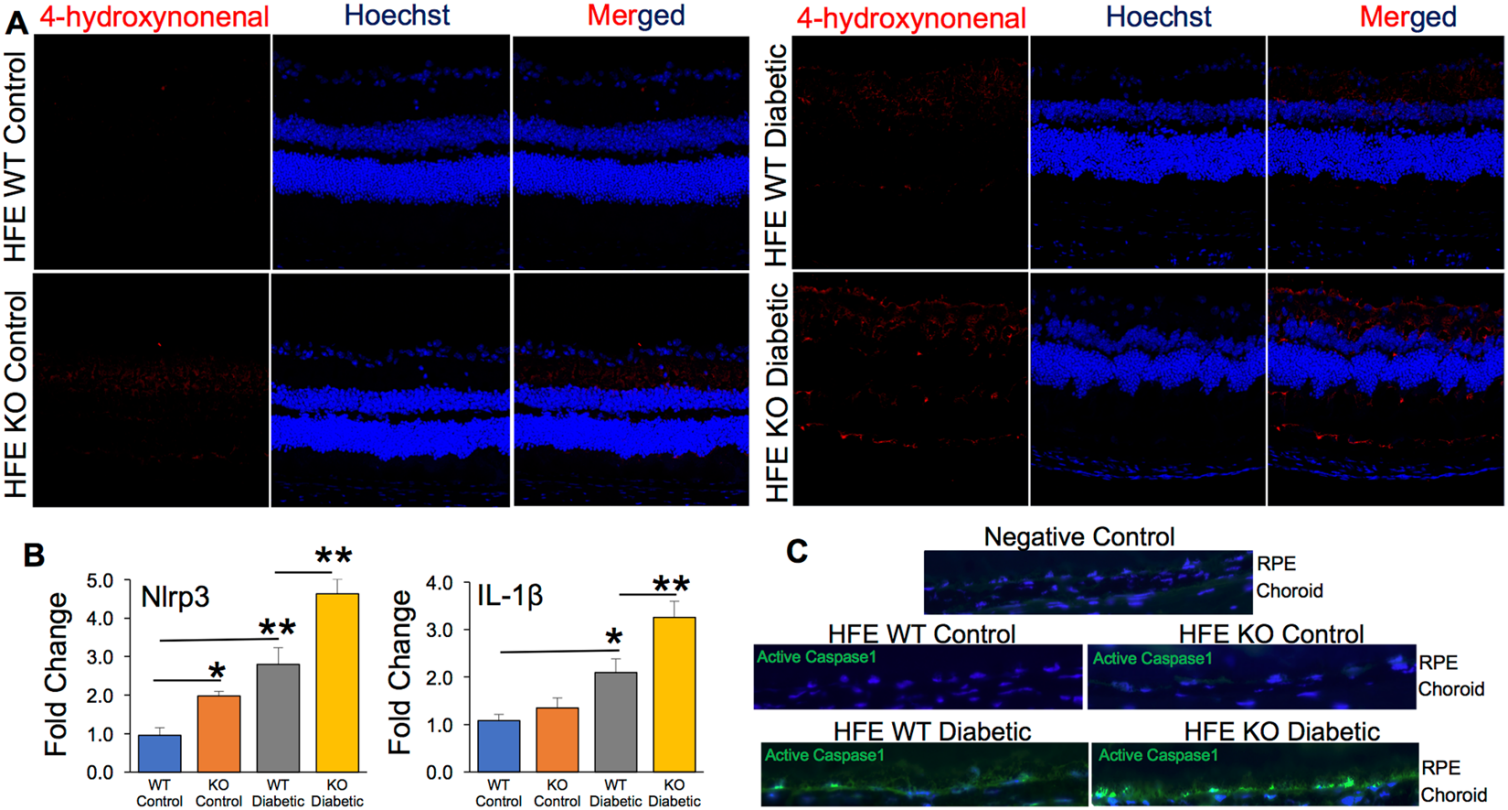
Increased oxidative stress and NLRP3 inflammasome activation in retina of diabetic HFE KO mouse model of iron overload. (**A**) 4-Hydroxynonenal staining in non-diabetic control and diabetic HFE WT and KO mouse retina. (**B**) Expression levels of NLRP3 and IL-1ß mRNA in non-diabetic control and diabetic HFE WT and KO mouse retina by RT-PCR (**C**) Active caspase-1 levels detected using the FAM-FLICA Assay Kit (*green*). Data are presented as mean ± S.E. *p < 0.01, **p < 0.001.

Oxidative stress and inflammation are postulated to promote diabetic retinopathy^39,40^. Animal studies have demonstrated that oxidative stress contributes to the development of DR as well as to the resistance of retinopathy to reverse after glycemic control is normalized^41^. Here we determined whether the expression of oxidative stress marker increased with retinal iron accumulation during diabetes. 4-hydroxynonenal (HNE), a lipid peroxidation product, is upregulated during oxidative stress^42^. Immunostaining of retinal cryosections for 4-HNE revealed mild fluorescence signal in retinal sections from non-diabetic HFE KO control in comparison to non-diabetic HFE WT control that did not have any positive fluorescence staining. Also, we found robust increase in the fluorescent intensity in diabetic HFE KO retinal sections compared to diabetic HFE WT sections (Fig. 5A). However, this effect was not surprising as it is known that iron overload promotes Fenton’s reaction, thereby increasing oxidative stress^9^. Labile iron was recently found to activate NLRP3 (NLR Family Pyrin Domain Containing 3) inflammasome signaling pathway and its downstream targets interleukin IL-1ß and active caspase-1^43,44^. To confirm these observations, we evaluated the expression of NLRP3 and IL-1ß by RT-PCR. We found increase in the expression levels of NLRP3 and IL-1ß in the retinas of diabetic HFE WT mice compared to non-diabetic HFE WT control mice, and the upregulation of NLRP3 and IL-1ß were more significant in diabetic HFE KO mice than diabetic HFE WT mice (Fig. 5B). Activated caspase-1, another downstream target of NLRP3 was also enhanced in diabetic HFE KO mice than in diabetic HFE WT mice with no detectable staining in the non-diabetic HFE WT and KO control (Fig. 5C) indicating that retinal iron overload during DR accelerates NLRP3 inflammasome signaling pathway. This is a significant finding considering a recent report showing NLRP3 inflammasome activation to be associated with the pathogenesis of proliferative diabetic retinopathy (PDR)^45^. Along with VEGF, the levels of caspase-1 and IL-18 were found to be predominantly increased in the vitreous samples of PDR patients. Interestingly, significantly higher levels of NLRP3 were present in the vitreous of PDR patients with tractional retinal detachment than in PDR patients with fully attached retina^45^.

### Retinal renin expression is induced through succinate receptor GPR91 in diabetic mice with iron overload

**Figure 6:**
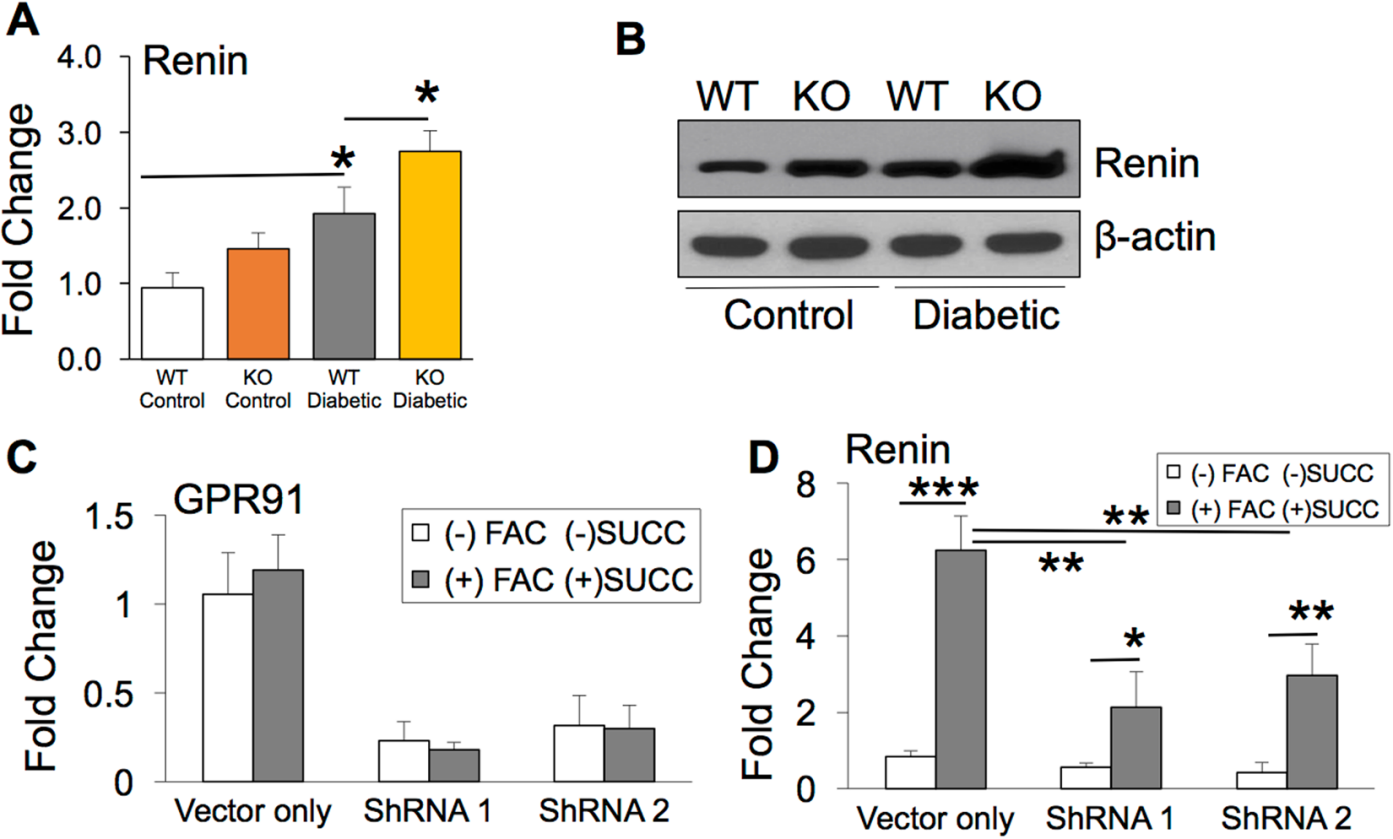
Iron overload during diabetic retinopathy upregulates retinal renin expression in a GPR91 dependent mechanism. (**A**) Pro/renin mRNA quantified in retina of non-diabetic and diabetic HFE WT and HFE KO mice by RT PCR (**B**) Renin protein levels quantified in retina of non-diabetic and diabetic HFE WT and HFE KO mice by western blot. Blots cropped from different parts of the same gel or from different gels are separated by white space. (**C**) Lentivirus-mediated knockdown of GPR91 expression in ARPE-19 cells treated with two different GPR91-specific shRNAs^34^. GPR91 knockdown was quantified by RT-PCR with 18S RNA as an internal control. Control ARPE-19 cells (vector only) and GPR91 shRNA-expressing ARPE-19 cells were treated with 100 μg/mL FAC for 72 hours, and during the last 16 hours of this 72-hour FAC treatment, cells were incubated with or without 2 mM succinate. (**D**) Renin mRNA levels were analyzed using RT-PCR in RNA from control ARPE-19 cells (vector only) and GPR91 shRNA-expressing ARPE-19 cells with or without the treatment of FAC and succinate. 18S RNA was used as an internal control. Data presented as mean ± SE of three experiments. *p < 0.05; **p < 0.001; ***p < 0.0001.

**Figure 7:**
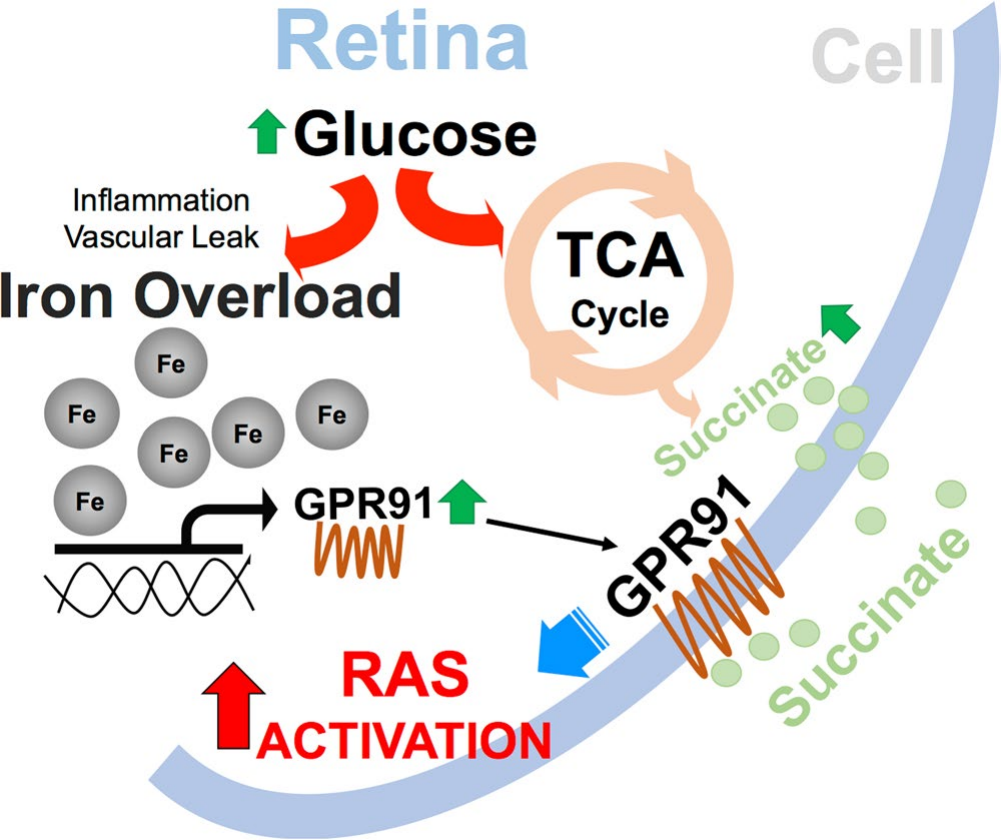
Illustration of the pathways linking iron and renin angiotensin system in diabetic retina. A schematic of the signaling pathways involved in the induction of retinal renin angiotensin system (RAS) during diabetes mediated by iron overload, GPR91 and succinate.

Succinate, an intermediate of glucose metabolism, has been shown to induce the expression of renal renin by signaling through its receptor GPR91^31,32^. We have reported previously that retinal iron overload enhances GPR91 expression in HFE and hemojuvelin knockout mouse models of hemochromatosis, a genetic disorder of iron overload^34,35^. In this study, we determined if retinal iron overload during DR activates GPR91 and thereby induces retinal renin expression. We found significant increase in retinal renin expression in the diabetic HFE KO mice compared to the diabetic HFE WT mice by RT-PCR and western blot (Figs. 6A and 6B). To determine if iron overload induces renin expression through GPR91 signaling, renin mRNA levels in ARPE-19 cells with normal expression of GPR91 was compared to GPR91 knockdown ARPE-19 cells, in the presence or absence of ferric ammonium citrate (FAC) and succinate. As reported in our previous publication^34^, among the four GPR91-specific shRNAs tested, two shRNAs (shRNA1 and shRNA2) showed significant knockdown of GPR91 expression^34^. We confirmed the GPR91 knockdown in cells treated with shRNA1 and 2 by Real time-PCR (Fig. 6C) before using the cells for the present study. Renin mRNA expression was upregulated in control ARPE-19 cells treated with FAC and succinate, while this upregulation in renin expression was significantly reduced in GPR91 knockdown ARPE-19 cells treated with FAC and succinate (Fig. 6D). These results indicate that renin expression in iron-overloaded RPE cells is induced by succinate-GPR91 signaling (Fig. 7).

## Discussion

There is a lack of understanding on the link between tissue iron and DR progression. The present study demonstrates that retina accumulates iron during diabetes. We have shown here for the first time that systemic iron overload during diabetes exacerbates the progression of DR in mice. We have a novel finding that during DR, retinal iron overload affects blood retinal barrier integrity and accelerates retinal cell loss by enhancing oxidative stress and NLRP3 inflammasome signaling. Another important outcome of the present study is that iron overload induces retinal renin expression through succinate receptor GPR91 signaling. The significance of this discovery is evident from many clinical reports demonstrating that renin-angiotensin system inhibitors delay DR progression^46^.

Increased oxidative stress and inflammation is considered a causal link between hyperglycemia and other metabolic anomalies involved in the pathogenesis of diabetic complications. Many clinical reports indicate that type 2 diabetic patients have elevated iron deposits and total body iron stores than non-diabetic control population^47–51^. Emerging studies suggest that excess body iron^52–54^ or high intake of dietary iron^55,56^ is related to the pathogenesis of diabetic complications. Strong evidences link iron to diabetic nephropathy with increased iron in diabetic animal kidneys^57,58^ and human urine^59^. Most importantly, low-iron diet or iron chelators have been shown to delay diabetic nephropathy progression^60,61^. The present study is the first report on the role of iron in the pathogenesis of diabetic retinopathy. During DR, the retinal microenvironment may accumulate iron by various mechanisms. Hyperglycemia has been reported to cause break down of heme containing molecules releasing free iron^62^. Intraretinal and intravitreal hemorrhages associated with DR also results in additional iron overload. Importantly, studies from our lab and others strongly suggest that inflammation can alter the expression of iron-regulatory gene hepcidin leading to iron accumulation^42,63^. On the contrary, diabetes is a common complication in patients with mutation in iron-regulatory proteins like hemochromatosis, and iron accumulation will exponentially increase during the progression of DR in these patients^19–21^. Thus our present study demonstrating iron accumulation in diabetic retina and the acceleration of DR progression with excess iron has high clinical significance.

DR is considered a microcirculatory disease of the retina triggered by hyperglycemia. However, studies from the past two decades indicate retinal neurodegeneration as an early event in the pathogenesis of DR occurring prior to the microcirculatory abnormalities in DR^64^. Therefore, the study of mechanisms that lead to neurodegeneration will help in identifying new therapeutic targets for the early stages of DR. Overexpression of retinal renin-angiotensin system has been shown to play a vital role in the neurodegeneration during DR^65–67^. The effects of renin-angiotensin system on retinal blood vessels are well established, causing vasoconstriction of retinal arterioles and capillaries^68^. In addition to vasoconstriction, RAS is known to modulate other functions such as endothelial cell migration, proliferation and angiogenic growth factor expression^69,70^. Diabetes has been shown to significantly induce plasma and renal prorenin levels by more than 20-fold in GPR91+/+ mice but this increase was greatly inhibited in diabetic GPR91−/− mice^31^. It has been shown recently that during hyperglycemia increase in succinate levels acts through GPR91 to trigger VEGF release in retinal ganglion cells by signaling extracellular signal-regulated kinase 1/2 (ERK1/2), p38 mitogen-activated protein kinase (p38 MAPK) and c-Jun N-terminal kinase (JNK)^33,71^. We have reported previously that retinal iron overload upregulates GPR91 expression and subsequently enhances the levels of pro-angiogenic factors like VEGF and angiopoietin in genetic mouse models of iron overload^34,35^. Here, we have shown that iron accumulation during diabetes upregulates retinal renin expression in a GPR91-dependent manner. The plasma and intrarenal renin is found to be activated early in diabetes; yet the exact mechanism is not known. This is the first study to demonstrate that iron overload regulates local renin expression.

Retina is constantly exposed to oxidative stress caused by photo-oxidation, and regulation of iron homeostasis within this tissue is important to prevent exacerbation of this oxidative stress by iron overload. The harmful effects of iron overload in causing inflammation and oxidative stress has been recognized for many decades. However, the results from this study identify a novel interaction between iron, GPR91 and RAS that plays an important role in neurodegeneration and vascular abnormalities associated with diabetes. Future experiments will be directed at using GPR91 knockout mouse model to understand the downstream pathways that are altered due to retinal renin overexpression during iron overload associated with DR. Without knowledge of retinal iron levels, intake of iron-rich diet or iron supplements by patients with diabetes may lead to a precarious situation. Hence it is critical to study the mechanisms by which iron contributes to retinal degeneration during DR. Further studies are warranted to confirm the effectiveness of iron chelators, GPR91 antagonists and renin-angiotensin inhibitors to prevent the exacerbation of DR progression caused by retinal iron overload. This knowledge will offer new avenues and targets for the treatment of not only diabetic retinopathy but also other vascular and neuronal disorders associated with iron overload.

## Materials and Methods

### Materials

Reagents were obtained from the following sources: reagent for RNA preparation, TRIzol (Invitrogen-Gibco Corp., Grand Island, NY), DMEM/F12 culture medium (Invitrogen-Gibco Corp., Grand Island, NY), RT-PCR kit (Applied Biosystems Inc., Foster City, CA), *Taq* polymerase kit (TaKaRa, Tokyo, Japan) and SyBr Green for Realtime PCR from Applied Biosystems Inc., (Foster City, CA). Rabbit polyclonal anti-4-hydroxynonenal (HNE) was from Alpha Diagnostic International (San Antonio, TX) and rabbit polyclonal anti zona occludens-1 (anti ZO-1) was from Abcam (Cambridge, MA). Rabbit anti-H-ferritin and rabbit anti-L-ferritin antibodies were a generous gift from Dr. Paolo Arosio, University of Brescia, Brescia, Italy. Goat anti-rabbit IgG coupled to Alexa Fluor 568 and goat anti-rabbit Alexa Fluor 488 were from Molecular Probes (Carlsbad, CA). FeRhoNox-1 fluorescent imaging probe was obtained from Goryo Chemical (Sapparo, Japan). Mouse ferritin ELISA kit was obtained from Kamiya Biomedical Company (Seattle, WA, U.S.A.).

### Animals

Breeding pairs of *HFE*^+/−^ mice were obtained from the Jackson Laboratory (Bar Harbor, ME). Normal male C57BL/6 mice were purchased from the Jackson Laboratory (Bar Harbor, ME) and used for induction of type 1 diabetes using intraperitoneal injection of streptozotocin per our previously published method^72^. For type 2 diabetes mice model, male BKS.Cg *Lepr*^db^/J (*db/db*) mice aged 12 weeks and age-matched wild-type (WT) C57BLKS/J mice were also obtained from Jackson Laboratory. Histologic processing of mouse eyes was performed following our previously published protocol^34^. All procedures involving mice were approved by the Augusta University and Saint Louis University Institutional Committee on Animal Use for Research and Education, and were performed in accordance with the Association for Research in Vision and Ophthalmology Statement for the Use of Animals in Ophthalmic and Vision Research.

### Postmortem human retina specimens

OCT frozen sections from normal and diabetic retinopathy patients were commercially obtained from National Development and Research Institute (NDRI) with permission of exemption from Augusta University Institutional Review Board (IRB). The Augusta University IRB Committee approved all procedures involved in tissue procurement from NDRI. The methods were carried out in accordance with the approved guidelines and regulations.

### Preparation of retinal tissues for immunostaining and RNA isolation

Mouse eyes were embedded in OCT compound and frozen at −80 °C. Sections (8-μm thick) were used for immunostaining. For preparation of RNA from posterior segments, corneas and lenses were removed using a stereomicroscope, and the posterior cup with retina was collected for RNA preparation.

### Real-time PCR

The following primers were used for quantification of mRNA by Real-time PCR: mouse NLRP3 forward primer 5′-TGCCTGTTCTTCCAGACTGGTGA-3′ and reverse primer 5′-CACAGCACCCTCATGCCCGG-3′; mouse IL-1β GCCCATCCTCTGTGACTCATAGGCCAC AGGTATTTTGTCG; mouse Pro/renin forward primer 5′-TCTGGGCACTCTTGTTGCTC-3′ and reverse primer 5′-GGGGGAGGTAAGATTGGTCAA-3′. Primers for human GPR91 were 5′-GGGGAGAAGAGAAGAGAAGAG-3′ (forward) and 5′-CACGGCATTGGACATGTA-3′ (reverse) and human renin were 5′-CCACCTCCTCCGTGATCCT-3′ (forward) and 5′-CGCCATAGTACTGGGTGTCCAT-3′ (reverse). Each PCR experiment was repeated at least three times with similar results. 18S mRNA was used as the internal control for PCR reaction. Real-time amplifications using detection chemistry (SYBR Green; Applied Biosystems), were run in triplicates on 96-well reaction plates using Step One Plus Real time PCR System.

### Immunofluorescence analysis

Retinal sections, fixed in 4% paraformaldehyde and blocked with 1× power block, were incubated overnight at 4 °C with one of the following primary antibodies: polyclonal rabbit anti-H-ferritin (1: 2,000), rabbit anti-L-ferritin (1: 2,000) or rabbit anti-HNE (1:500 dilution). Negative controls involved omission of the primary antibodies. Sections were rinsed and incubated for 1 hour with goat anti-rabbit IgG coupled to Alexa Fluor 568. Active caspase-1levels detected using the FAM-FLICA Assay Kit. Coverslips were mounted after staining with Hoechst nuclear stain, and sections were examined by laser-scanning confocal microscopy (Carl Zeiss, Oberkochen, Germany). Immunohistochemistry studies were repeated twice, and the results from these two experiments were similar.

### Histological detection of labile Fe(II)

Labile iron (Fe^2+^) in retinal sections were detected using FeRhoNox-1 fluorescent imaging probe as mentioned in publications^73,74^. RhoNox-1 was dissolved in dimethyl sulfoxide to produce a 1 mM solution, which was further diluted to a final concentration of 5 μM. This diluted solution was prepared fresh just before use. Frozen retinal sections of 8 μm thickness were air dried for 3 min, fixed in 4% paraformaldehyde for 1 min, and washed in deionized water briefly. Then, 200 μl of 5 μM RhoNox-1 was placed on the retinal sections and incubated for 30 min at 37 °C in a dark chamber. The sections were then counterstained with Hoechst nuclear stain and observed using laser-scanning confocal microscopy.

### Western blot analysis

Protein was extracted from retinal tissues and concentration was determined using the bicinchoninic acid (BCA) assay (Thermo Fisher Scientific, Rockford, IL). 20 μg of protein were subjected to SDS–PAGE, transferred to nitrocellulose membranes, and then incubated with primary antibodies overnight at 4 °C. Secondary detection was done using horseradish peroxidase– conjugated secondary antibodies (Promega, Madison, WI). After washing, the proteins were visualized using enhanced chemiluminescence (ECL) western blot detection system (Thermo Fisher Scientific). β- actin served as the loading control. Western blot analysis was repeated twice with comparable results.

### Enzyme-linked immunosorbent assay

A mouse ferritin ELISA kit (mentioned in Materials) was used for quantification of ferritin levels in mouse serum and retinas as published previously^75^. A calibration curve was used as instructed in the kit to determine ferritin levels. ELISA plates were read spectrophotometrically at 450 nm using a SpectraMAX 190 plate reader (Molecular Devices, Sunnyvale, CA).

### Measurement of reactive oxygen species

Excessive iron accumulation in cells generates hydroxyl radicals via the Fenton reaction, which then promote lipid peroxidation. 4-Hydroxynonenal is one of the by-products of lipid peroxidation and is widely used as a marker of oxidative stress. Retinal sections were stained with anti-HNE antibody as per our published method^76^.

### *In situ* detection of DNA fragmentation by TUNEL assay

TUNEL assay was performed using the ApopTAG fluorescein *in situ* apoptosis detection kit. After staining, the tissue sections were viewed by epifluorescence using standard fluorescence excitation and emission filters. Each section was scanned systematically from the temporal to the nasal ora serrata for fluorescent cells indicative of apoptosis. To distinguish between structures that autofluoresced versus those that were TUNEL-positive, all slides were examined first with the rhodamine filter and then with the FITC filter. Autofluorescent structures were visible under both filters, whereas TUNEL-positive cells were detectable only with the FITC filter.

### RPE flat mount

RPE whole mount was prepared as described by Longbottom *et al*.^77^. In brief, RPE/eyecups were dissected from unfixed eyes and then fixed in ice cold methanol for at least overnight, and processed for immunohistochemistry. The fixed RPE/eyecups were blocked with 0.1% BSA, 2% goat serum, and 0.1% Triton-X in PBS for 1 hour. Subsequently, the RPE/eyecups were incubated with a rabbit polyclonal antibody against ZO-1 (1:100) diluted in the blocking solution overnight at 4 °C, and then with goat anti rabbit Alexa Fluor 488 secondary antibody for 1 hour at room temperature. RPE/eyecups were then cut partially at four places to allow the tissue to be flattened upon Superfrost microscope slides (Fisher Scientific, Pittsburgh, PA) and viewed by laser-scanning confocal microscopy.

### Establishment of mouse primary RPE cell cultures

Age-matched WT and HFE KO mice were obtained from the same litter originating from the mating of heterozygous mice. Three-week-old mice were used to establish primary cultures of RPE as described previously^42^.

### Treatment of RPE cells with ferric ammonium citrate

Primary RPE cells and human RPE cell lines ARPE-19 and HRPE were seeded in 24-well culture plates and cultured for 24 hours. Fresh culture medium was then added to cells with or without ferric ammonium citrate (FAC) (100 μg/mL), and the cells were cultured for an additional 72 hours. Cells were then used for RNA extraction. The concentration of FAC and the treatment time were selected based on published reports to cause iron overload in cells for optimal iron-induced gene expression changes^36^.

### Transepithelial permeability assay

HFE WT and KO primary RPE cells were seeded on non-coated membranes with 0.4 *μ*m pores (Transwell; Corning Costar), in Dulbecco’s modified Eagle’s medium: nutrient mixture F-12 (DMEM/F-12). After becoming completely confluent, FITC- dextran (40 kDa; Sigma-Aldrich) was then added to the upper chambers and samples from the lower and upper chambers were obtained at different time points. The concentration of FITC-dextran in these samples was quantified by microplate reader (ELx800; Bio-Tek). The diffusion rate was calculated as (amount of dextran lower chamber)/(amount of dextran upper chamber) and expressed as relative quantification. Each experiment was repeated four times.

### Fundus and angiography analyses

To evaluate retinal integrity as well as vasculature and permeability *in vivo*, mice were anesthetized using a 20 µl intramuscular injection of rodent anesthesia cocktail (ketamine 100 mg/mL, xylazine 30 mg/mL, acepromazine 10 mg/mL). Pupils were dilated using 1% tropicamide (Bausch and Lomb, Rochester, NY). The mouse was placed on the imaging platform of the Phoenix Micron III retinal imaging microscope (Phoenix Research Laboratories, Pleasanton, CA), and Goniovisc 2.5% (hypromellose; Sigma Pharmaceuticals, LLC, Monticello, IA) was applied liberally to keep the eye moist during imaging. For angiography analysis, mice were administered 10 to 20 μl fluorescein sodium (10% Lite) (Apollo Ophthalmics, Newport Beach, CA) (while also receiving Goniovisc 2.5% [hypromellose; Sigma Pharmaceuticals, LLC]), and rapid acquisition of fluorescent images ensued for approximately 5 minutes as mentioned in our previous publication^78^.

### shRNA-induced silencing of GPR91 in ARPE-19 cells

Lentiviral-based shRNAs were used to knock down GPR91 in ARPE-19 cells as published previously^34^. We generated stable cell lines of ARPE-19 with GPR91 knockdown using gene-specific shRNA constructs with the lentivirus plasmid vector pLKO.1-puromycin (Open Biosystems, Huntsville, AL). The following two constructs which had maximum knockdown^34^ were used: shRNA1 (clone TRCN0000008976) and shRNA2 (clone TRCN0000008977). Stable cell lines were generated by infecting ARPE-19 cells independently with one of the two lentiviral-shRNA constructs and then selecting stable cell lines by puromycin (1 μg/mL) resistance. A stable cell line with a lentiviral vector only, without the shRNA, was used as the negative control. Knockdown of GPR91 expression was verified by RT-PCR. The sequences of shRNAs are as follows: CCGGGCCTCTCAACTTGGTCATCATCTCGAGATGATGACCAAGTTGAG AGGCTTTTT (shRNA1); CCGGCGGCTACATCTTCTCTCTGAACTCGAGTTCAGAGA GAAGATGTAGCCGTTTTT (shRNA2).

### Data analysis

All experiments were repeated three to five times with independent cell or tissue preparations and samples run in duplicate. Data are presented as mean ± standard error of the mean (SEM). Statistical significance was determined with the Student *t* test, and one- way ANOVA with Tukey–Kramer’s post-hoc tests for comparisons between two groups or multiple groups, respectively. Differences were considered statistically significant at p < 0.05.

### Data availability statement

The datasets generated and analyzed during the current study are available from the corresponding author on reasonable request.

## Electronic supplementary material

Supplementary Information Western blots

## References

[1] Congdon NG, Friedman DS, Lietman T (2003). Important causes of visual impairment in the world today. Journal of the American Medical Association.

[2] Tang J, Kern TS (2011). Inflammation in diabetic retinopathy. Prog Retin Eye Res..

[3] Barber AJ (1998). Neural apoptosis in the retina during experimental and human diabetes: early onset and effect of insulin. Journal of Clinical Investigation.

[4] Antonetti DA (2006). Diabetic retinopathy: seeing beyond glucose-induced microvascular disease. Diabetes.

[5] Simo R, Carrasco E, Garcıa-Ramırez M, Hernandez C (2006). Angiogenic and antiangiogenic factors in proliferative diabetic retinopathy. Current Diabetes Reviews.

[6] Klein R (1984). The Wisconsin epidemiologic study of diabetic retinopathy II. Prevalence and risk of diabetic retinopathy when age at diagnosis is less than 30 years. Arch Ophthalmol..

[7] Klein R (1984). The Wisconsin epidemiologic study of diabetic retinopathy when age at diagnosis is 30 or more years. Arch Ophthalmol..

[8] Madsen-Bouterse SA, Kowluru RA (2008). Oxidative stress and diabetic retinopathy: pathophysiological mechanisms and treatment perspectives. Reviews in Endocrine and Metabolic Disorders.

[9] Halliwell B, Gutteridge JM (1984). Oxygen toxicity, oxygen radicals, transition metals and disease. Biochem J..

[10] Hare DJ (2015). Is early-life iron exposure critical in neurodegeneration?. Nat Rev Neurol..

[11] Schröder N, Figueiredo LS, de Lima MN (2013). Role of brain iron accumulation in cognitive dysfunction: evidence from animal models and human studies. J Alzheimers Dis..

[12] Greenough MA, Ramírez Munoz A, Bush AI, Opazo CM (2016). Metallo-pathways to Alzheimer’s disease: lessons from genetic disorders of copper trafficking. Metallomics.

[13] Wong RW, Richa DC, Hahn P, Green WR, Dunaief JL (2007). Iron toxicity as a potential factor in AMD. Retina.

[14] Song D (2016). AMD-like retinopathy associated with intravenous iron. Exp Eye Res..

[15] Dawczynski J, Blum M, Winnefeld K, Strobel J (2002). Increased content of zinc and iron in human cataractous lenses. Biological Trace Element Research.

[16] Farkas RH (2004). Increased expression of iron-regulating genes in monkey and human glaucoma. Investigative Ophthalmology and Visual Science.

[17] Konerirajapuram NS (2004). Trace elements iron, copper and zinc in vitreous of patients with various vitreoretinal diseases. Indian J Ophthalmol..

[18] Weller M, Clausen R, Heimann K, Wiedemann P (1990). Iron-binding proteins in the human vitreous: lactoferrin and transferrin in health and in proliferative intraocular disorders. Ophthalmic Res..

[19] Galton DJ (1965). Diabetic retinopathy and hemochromatosis. Br Med J..

[20] Walsh CH, Malins JM (1978). Proliferative retinopathy in a patient with diabetes mellitus and idiopathic haemochromatosis. Br Med J..

[21] Peterlin B, Globocnik Petrovic M, Makuc J, Hawlina M, Petrovic D (2003). A hemochromatosis-causing mutation C282Y is a risk factor for proliferative diabetic retinopathy in Caucasians with type 2 diabetes. J Hum Genet.

[22] Ciudin, A., Hernández, C. & Simó, R. Iron overload in diabetic retinopathy: a cause or a consequence of impaired mechanisms? *Exp Diabetes Res*. pii: 714108 (2010). 10.1155/2010/714108 PMC2935195 20827392

[23] Kobori H, Nangaku M, Navar LG, Nishiyama A (2007). The intrarenal renin-angiotensin system: from physiology to the pathobiology of hypertension and kidney disease. Pharmacol Rev..

[24] Wagner J (1996). Demonstration of renin mRNA, angiotensinogen mRNA, and angiotensin converting enzyme mRNA expression in the human eye: evidence for an intraocular renin-angiotensin system. Br J Ophthalmol..

[25] Nagai N (2007). Suppression of diabetes-induced retinal inflammation by blocking the angiotensin II type 1 receptor or its downstream nuclear factor-kappaB pathway. Invest Ophthalmol Vis Sci..

[26] Chaturvedi N (2008). Direct Programme Study Group. Effect of candesartan on prevention (Direct-Prevent 1) and progression (DIRECT-Project 1) of retinopathy in type 1 diabetes: randomised, placebo-controlled trials. Lancet.

[27] Sjolie AK (2008). Direct Programme Study Group Effect of candesartan on progression and regression of retinopathy in type 2 diabetes (Direct-Protect 2): a randomised placebo-controlled trial. Lancet.

[28] Mauer M (2009). Renal and retinal effects of enalapril and losartan in type 1 diabetes. N Engl J Med..

[29] He W (2004). Citric acid cycle intermediates as ligands for orphan G-protein-coupled receptors. Nature.

[30] Hebert SC (2004). Physiology: orphan detectors of metabolism. Nature.

[31] Toma I (2008). Succinate receptor GPR91 provides a direct link between high glucose levels and renin release in murine and rabbit kidney. J Clin Invest..

[32] Vargas SL, Toma I, Kang JJ, Meer EJ, Peti-Peterdi J (2009). Activation of the succinate receptor GPR91 in macula densa cells causes renin release. J Am Soc Nephrol..

[33] Sapieha P (2008). The succinate receptor GPR91 in neurons has a major role in retinal angiogenesis. Nat Med..

[34] Gnana-Prakasam JP (2011). Expression and Iron-Dependent Regulation of Succinate Receptor GPR91 in Retinal Pigment Epithelium. Invest Ophthalmol Vis Sci..

[35] Arjunan P (2016). Increased Retinal Expression of the Pro-Angiogenic Receptor GPR91 via BMP6 in a Mouse Model of Juvenile Hemochromatosis. Invest Ophthalmol Vis Sci..

[36] Gnana-Prakasam JP (2009). Absence of Iron-regulatory Protein HFE Results in Hyper-proliferation of Retinal Pigment Epithelium Mediated by Induction of Cystine/Glutamate Transporter. Biochem J..

[37] Gnana-Prakasam JP (2013). Loss of HFE Leads to Progression of Tumor Phenotype in Primary Retinal Pigment Epithelial Cells. Invest Ophthalmol Vis Sci..

[38] Cunha-Vaz J, Bernardes R, Lobo C (2011). Blood-retinal barrier. Eur J Ophthalmol..

[39] Vikram, A., Tripathi, D. N., Kumar, A. & Singh, S. Oxidative stress and inflammation in diabetic complications. *Int J Endocrinol*. 679754 (2014). 10.1155/2014/679754 PMC4026976 24876838

[40] Baynes JW, Thorpe SR (1999). Role of oxidative stress in diabetic complications: a new perspective on an old paradigm. Diabetes.

[41] Kowluru RA (2003). Effect of reinstitution of good glycemic control on retinal oxidative stress and nitrative stress in diabetic rats. Diabetes.

[42] Gnana-Prakasam JP (2008). Hepcidin expression in mouse retina and its regulation via lipopolysaccharide/ toll-like receptor-4 pathway independent of Hfe. Biochem J..

[43] Nakamura K (2016). Activation of the NLRP3 inflammasome by cellular labile iron. Exp Hematol..

[44] Gelfand BD (2015). Iron toxicity in the retina requires Alu RNA and the NLRP3 inflammasome. Cell Rep..

[45] Loukovaara, S. *et al*. NLRP3 inflammasome activation is associated with proliferative diabetic retinopathy. *Acta Ophthalmol*. Epub ahead of print (2017). 10.1111/aos.13427 28271611

[46] Wang B (2015). Effects of RAS inhibitors on diabetic retinopathy: a systematic review and meta-analysis. Lancet Diabetes Endocrinol..

[47] Jiang R (2004). Body iron stores in relation to risk of type 2 diabetes in apparently healthy women. J Am Med Assoc..

[48] Bao W, Rong Y, Rong S, Liu L (2012). Dietary iron intake, body iron stores, and the risk of type 2 diabetes: a systematic review and metaanalysis. BMC Med..

[49] Ford ES, Cogswell ME (1999). Diabetes and serum ferritin concentration among US adults. Diabetes Care.

[50] Eshed I, Elis A, Lishner M (2001). Plasma ferritin and type 2 diabetes mellitus: a critical review. Endocr Res.

[51] Arredondo M (2011). Cross-talk between body iron stores and diabetes: iron stores are associated with activity and microsatellite polymorphism of the heme oxygenase and type 2 diabetes. Biol Trace Elem Res..

[52] Wolff SP (1993). Diabetes mellitus and free radicals. Free radicals, transition metals and oxidative stress in the etiology of diabetes mellitus and complications. Br Med Bull.

[53] Swaminathan S, Fonseca VA, Alam MG, Shah SV (2007). The role of iron in diabetes and its complications. Diabetes Care.

[54] Li X (2012). Iron increases liver injury through oxidative/nitrative stress in diabetic rats: involvement of nitrotyrosination of glucokinase. Biochimie.

[55] Fung TT (2004). Dietary patterns, meat intake, and the risk of type 2 diabetes in women. Arch Intern Med..

[56] Li H (2008). Body iron stores and dietary iron intake in relation to diabetes in adults in North China. Diabetes Care.

[57] Dominguez JH, Liu Y, Kelly KJ (2015). Renal iron overload in rats with diabetic nephropathy. Physiol Rep.

[58] Ward DT (2005). Altered expression of iron transport proteins in streptozotocin-induced diabetic rat kidney. Biochim Biophys Acta..

[59] Howard RL, Buddington B, Alfrey AC (1991). Urinary albumin, transferrin and iron excretion in diabetic patients. Kidney Intl..

[60] Remuzzi A, Puntorieri S, Brugnetti B, Bertani T, Remuzzi G (1991). Renoprotective effect of low iron diet and its consequence on glomerular hemodynamics. Kidney Int..

[61] Nath KA, Fischereder M, Hostetter TH (1994). The role of oxidants in progressive renal injury. Kidney Int..

[62] Cussimanio BL, Booth AA, Todd P, Hudson BG, Khalifah RG (2003). Unusual susceptibility of heme proteins to damage by glucose during non-enzymatic glycation. Biophysical Chemistry.

[63] Wessling-Resnick M (2010). Iron Homeostasis and the Inflammatory Response. Annu Rev Nutr..

[64] Villarroel M, Ciudin A, Hernández C, Simó R (2010). Neurodegeneration: An early event of diabetic retinopathy. World J Diabetes.

[65] Silva KC, Rosales MA, Biswas SK, Lopes de Faria JB, Lopes de Faria JM (2009). Diabetic retinal neurodegeneration is associated with mitochondrial oxidative stress and is improved by an angiotensin receptor blocker in a model combining hypertension and diabetes. Diabetes.

[66] Kurihara T (2008). Angiotensin II type 1 receptor signaling contributes to synaptophysin degradation and neuronal dysfunction in the diabetic retina. Diabetes.

[67] Ozawa Y, Yuki K, Yamagishi R, Tsubota K, Aihara M (2013). Renin-angiotensin system involvement in the oxidative stress-induced neurodegeneration of cultured retinal ganglion cells. Jpn J Ophthalmol..

[68] Kawamura H (2004). Effects of angiotensin II on the pericyte containing microvasculature of the rat retina. J Physiol..

[69] Otani A, Takagi H, Suzuma K, Honda Y (1998). Angiotensin II potentiates vascular endothelial growth factor-induced angiogenic activity in retinal microcapillary endothelial cells. Circ Res..

[70] Otani A (2000). Angiotensin II-stimulated vascular endothelial growth factor expression in bovine retinal pericytes. Invest Ophthalmol Vis Sci..

[71] Hu J, Wu Q, Li T, Chen Y, Wang S (2013). Inhibition of high glucose-induced VEGF release in retinal ganglion cells by RNA interference targeting G protein-coupled receptor 91. Exp Eye Res..

[72] Gambhir D (2012). GPR109A as an anti-inflammatory receptor in retinal pigment epithelial cells and its relevance to diabetic retinopathy. Invest. Ophthalmol. Vis. Sci..

[73] Mukaide T (2014). Histological detection of catalytic ferrous iron with the selective turn-on fluorescent probe RhoNox-1 in a Fenton reaction-based rat renal carcinogenesis model. Free Radic Res..

[74] Wang Y (2016). Role of hemoglobin and transferrin in multi-wall carbon nanotube-induced mesothelial injury and carcinogenesis. Cancer Sci..

[75] Gnana-Prakasam JP (2012). Iron-mediated Retinal Degeneration in Hemojuvelin Knockout Mice. Biochem J..

[76] Gnana-Prakasam JP, Zhang M, Atherton SS, Smith SB, Ganapathy V (2009). Expression of Iron Regulatory Protein Hemojuvelin in Retina and its Potential Role in Cytomegalovirus induced Retinitis. Biochem J..

[77] Longbottom R (2009). Genetic ablation of retinal pigment epithelial cells reveals the adaptive response of the epithelium and impact on photoreceptors. Proc Natl Acad Sci USA.

[78] Tawfik A, Gnana-Prakasam JP, Smith SB, Ganapathy V (2014). Deletion of hemojuvelin, an iron-regulatory protein, in mice results in abnormal angiogenesis and vasculogenesis in retina along with reactive gliosis. Invest Ophthalmol Vis Sci..

