# Supplementary material for: Iron Overload Accelerates the Progression of Diabetic Retinopathy in Association with Increased Retinal Renin Expression
Source: Sci Rep. 2018 Feb 14;8:3025. doi: 10.1038/s41598-018-21276-2 (PMC5813018; doi:10.1038/s41598-018-21276-2)
Supplement: Supplementary file 1 — Supplementary Information Western blots [file 41598_2018_21276_MOESM1_ESM.pdf]

Iron Overload Accelerates the Progression of Diabetic Retinopathy in Association with Increased Retinal Renin Expression

Kapil Chaudhary[1], Wanwisa Promsote[2], Sudha Ananth[3], Rajalakshmi Veeranan-Karmegam[3], Amany Tawfik[4], Pachiappan Arjunan[4], Pamela Martin[3], Sylvia B. Smith[5], Muthusamy Thangaraju[3], Oleg Kisselev[7], Vadivel Ganapathy[6], Jaya P. Gnana-Prakasam[7]*

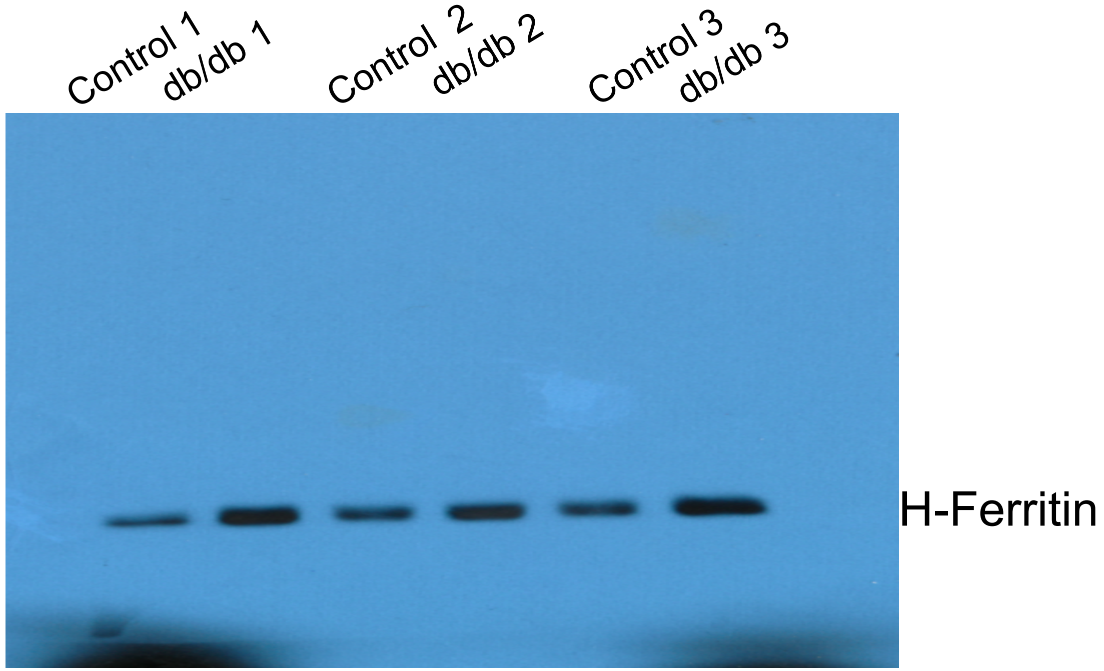
Control 1
db/db 1
Control 2
db/db 2
Control 3
db/db 3
H-Ferritin

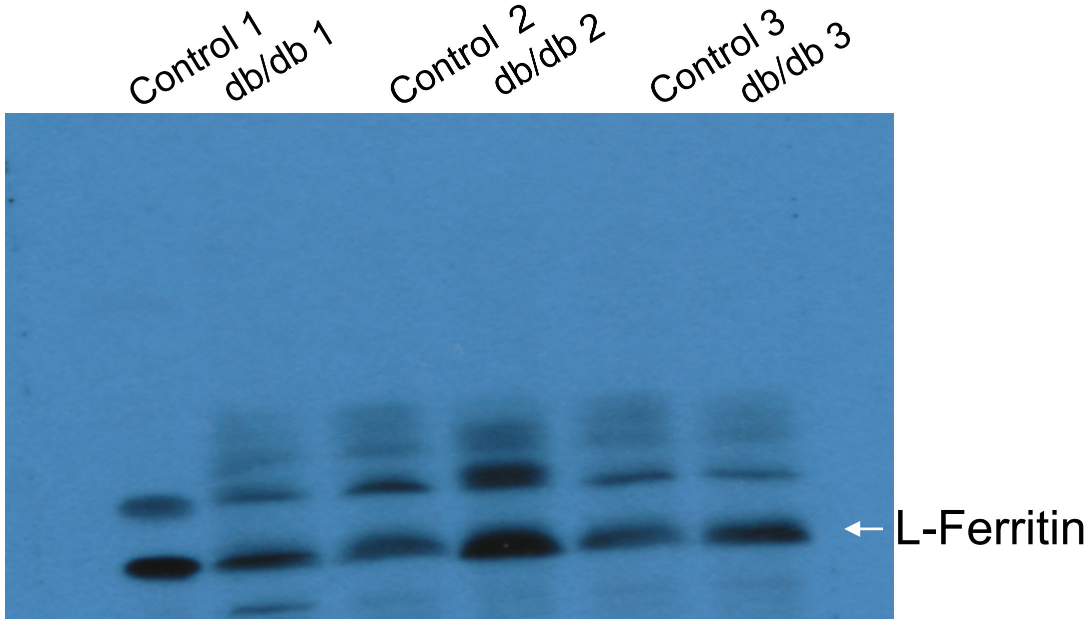
Control 1
db/db 1
Control 2
db/db 2
Control 3
db/db 3
L-Ferritin

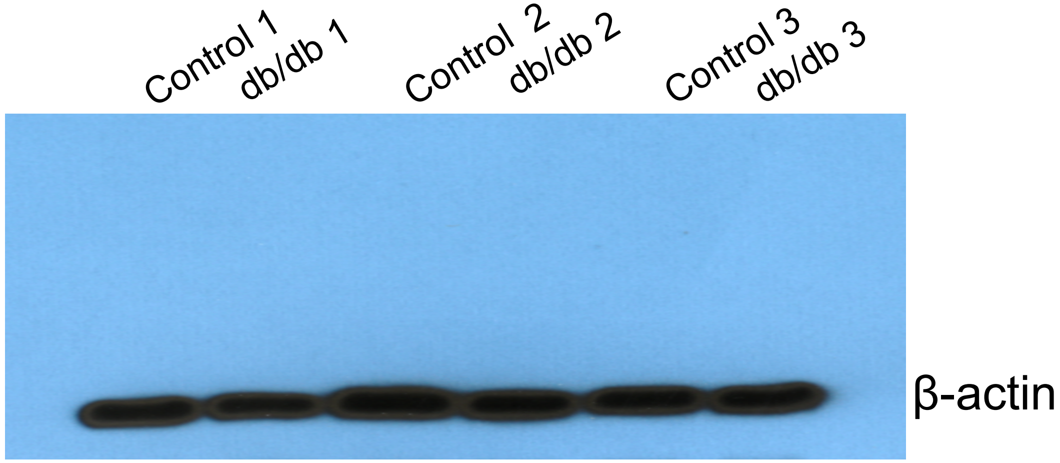
Control 1
db/db 1
Control 2
db/db 2
Control 3
db/db 3
β-actin

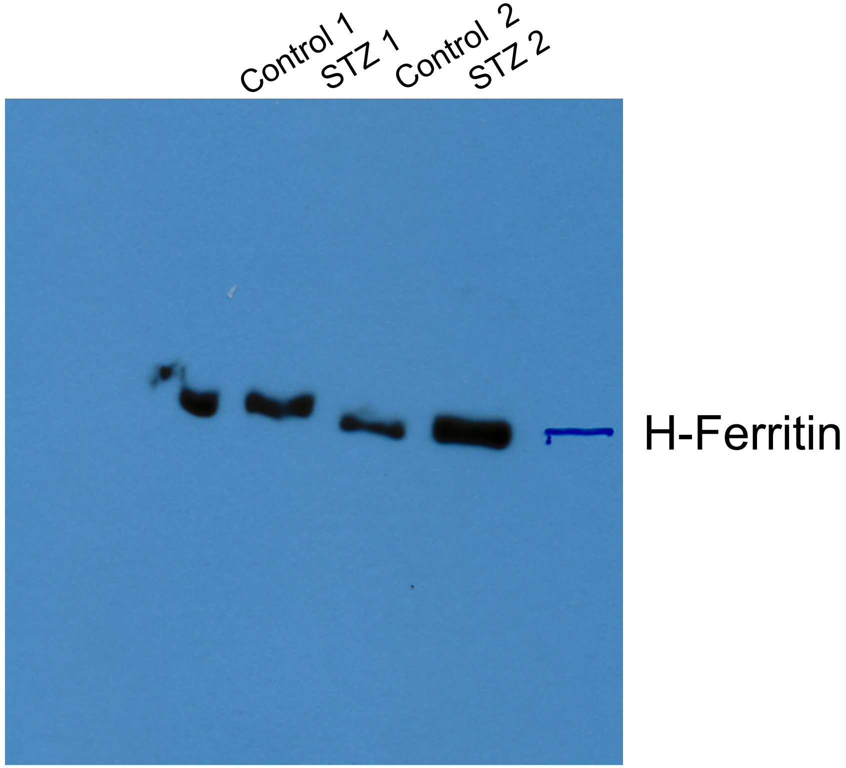
Control 1
STZ 1
Control 2
STZ 2
H-Ferritin

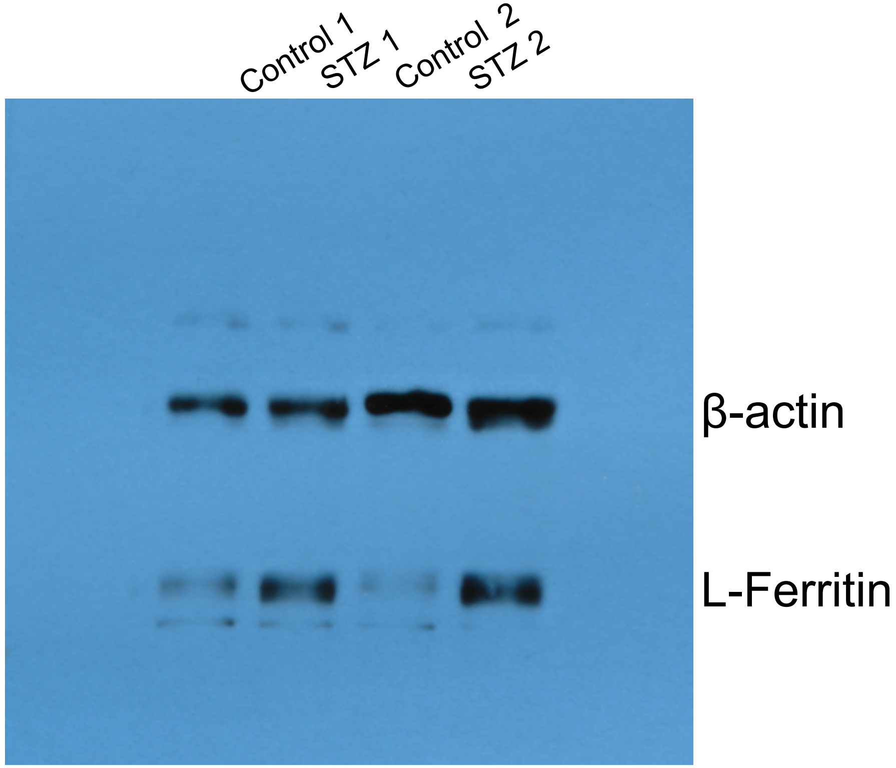
Control 1
STZ 1
Control 2
STZ 2
β-actin
L-Ferritin

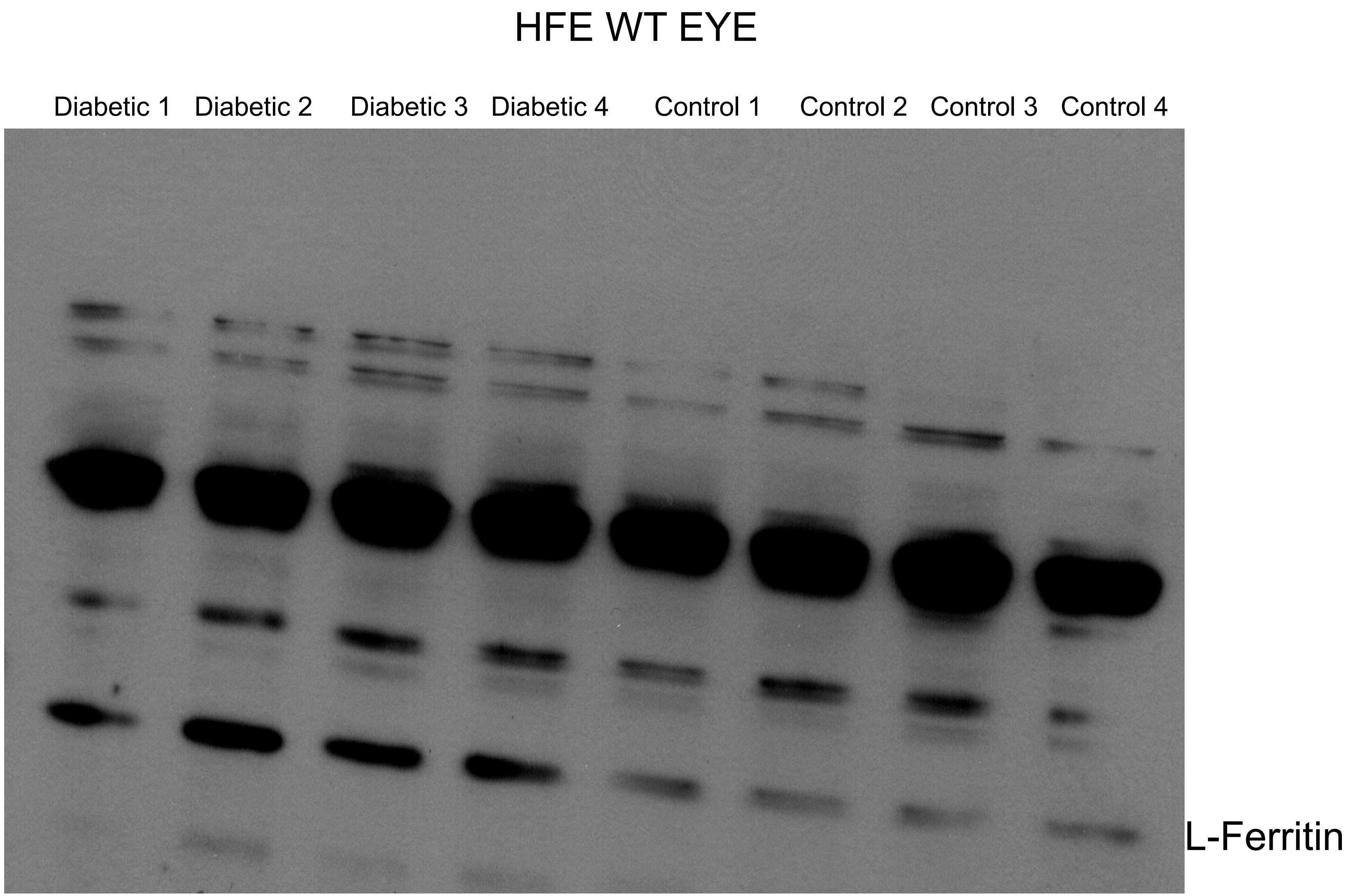
HFE WT EYE
Diabetic 1
Diabetic 2
Diabetic 3
Diabetic 4
Control 1
Control 2
Control 3
Control 4
L-Ferritin

HFE KO EYE

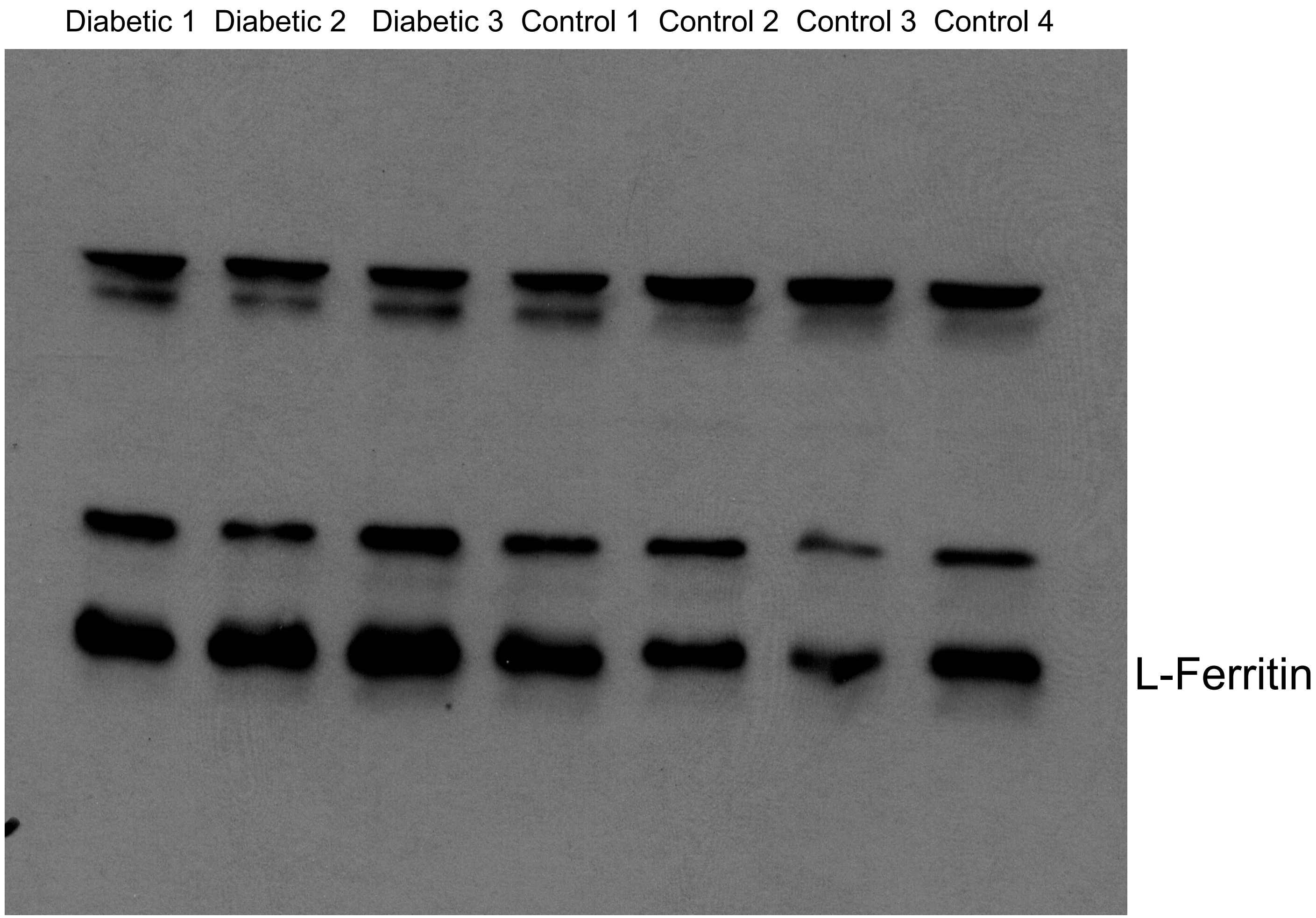
Diabetic 1
Diabetic 2
Diabetic 3
Control 1
Control 2
Control 3
Control 4
L-Ferritin

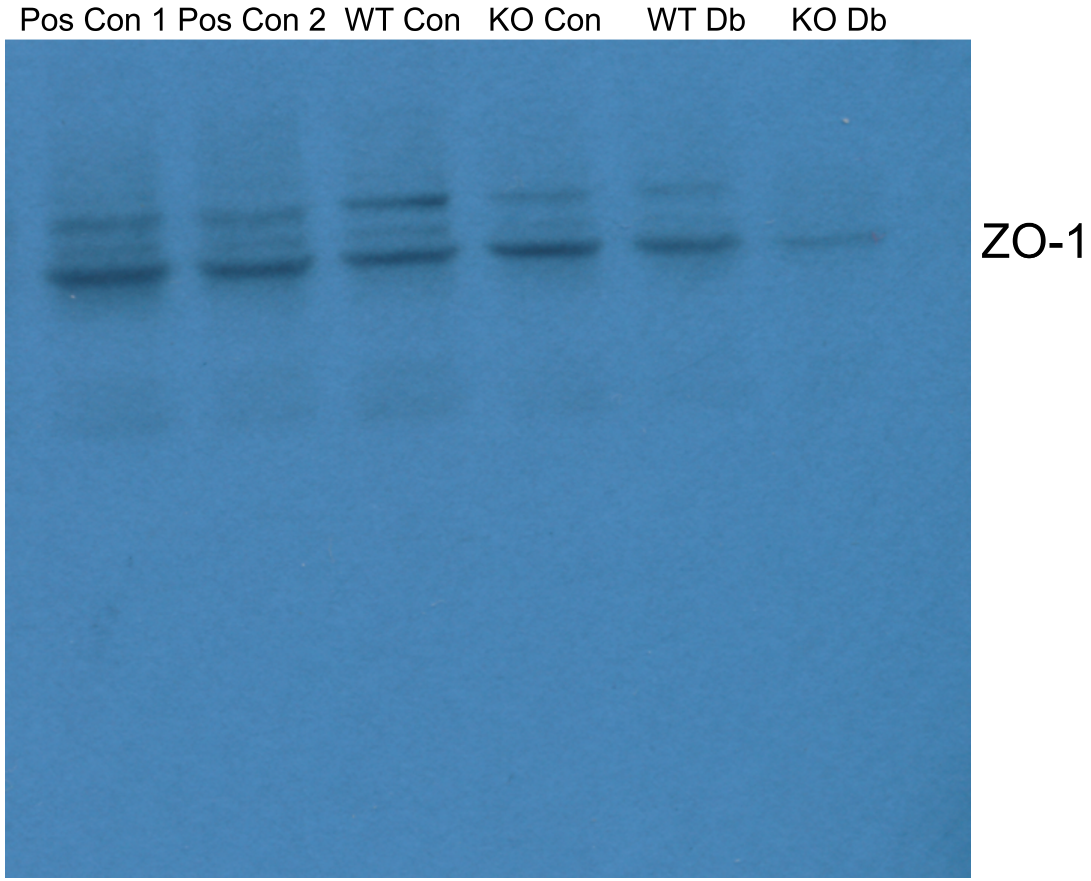
Pos Con 1
Pos Con 2
WT Con
KO Con
WT Db
KO Db
ZO-1

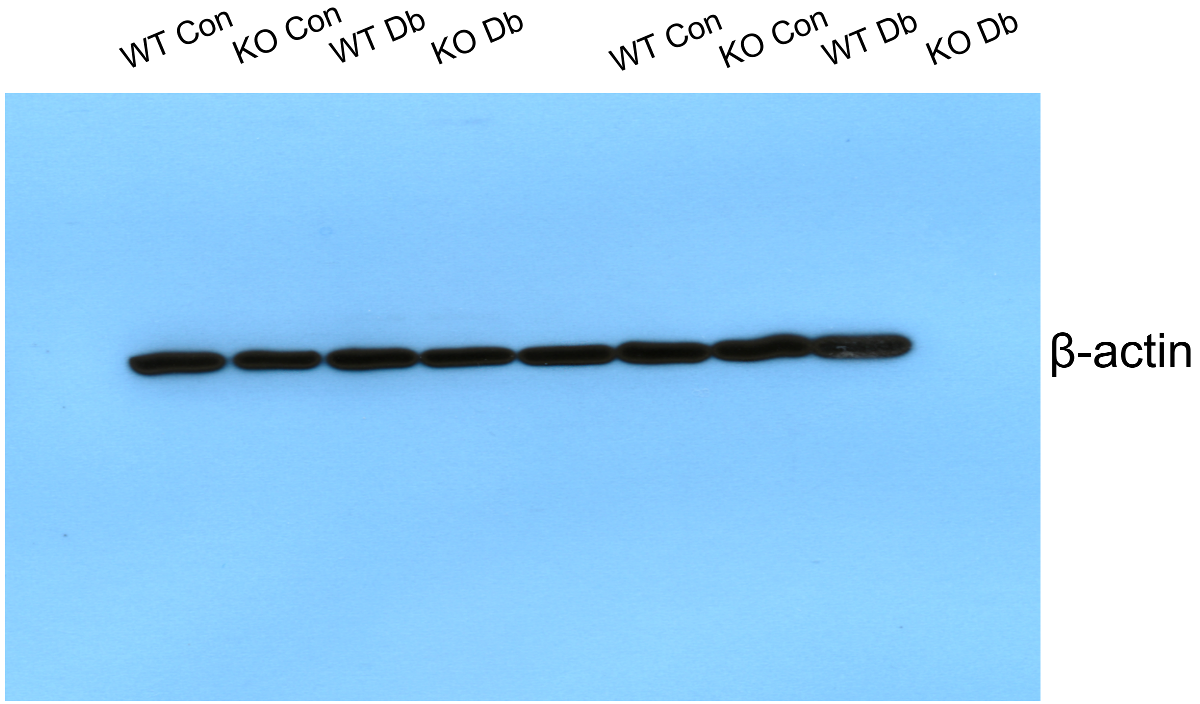
WT Con
KO Con
WT Db
KO Db
WT Con
KO Con
WT Db
KO Db
β-actin

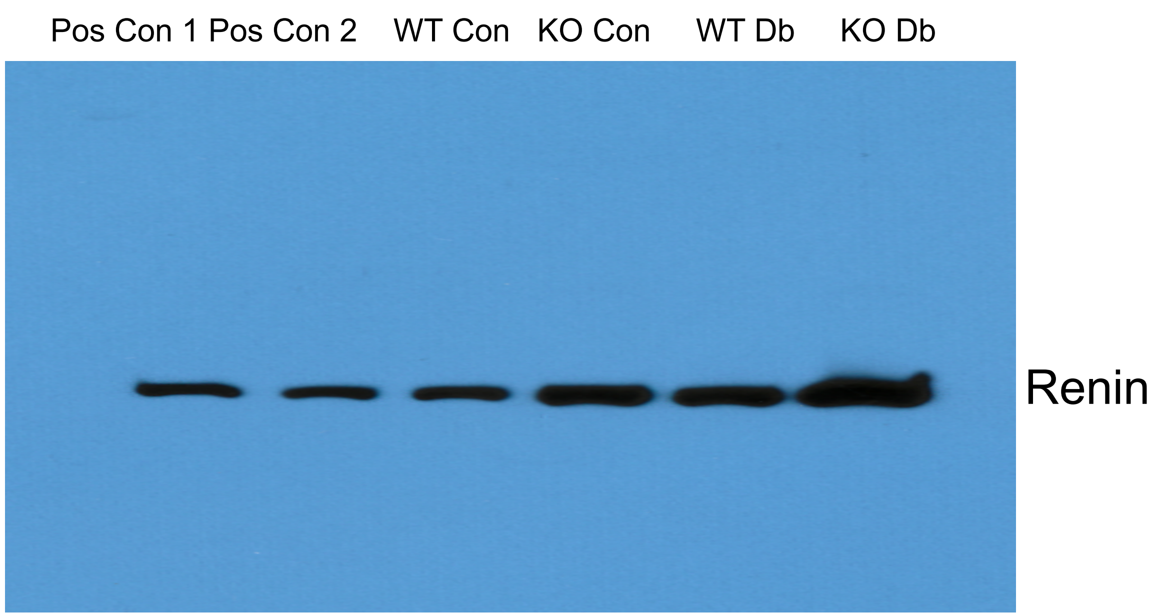
Pos Con 1
Pos Con 2
WT Con
KO Con
WT Db
KO Db
Renin
